# Peroneal Nerve Palsy After a Minimally Displaced Weber B Fibula Fracture: A Rare Case of Extensor Retinaculum Syndrome

**DOI:** 10.1177/24730114251387694

**Published:** 2025-11-14

**Authors:** William Collins, Nikolas Drobetz, Cyrus Mehta

**Affiliations:** 1 University of Sydney, Camperdown, NSW, Australia; 2 Department of Orthopaedics, Lismore Base Hospital, NSW, Australia

**Keywords:** ankle fracture, extensor retinaculum, superior extensor retinaculum, syndrome

## Introduction

Extensor retinaculum syndrome (ERS) is a rare presentation, similar to that of compartment syndrome, characterised clinically by severe pain, sensory disturbance of the deep peroneal nerve (DPN) disruption, weakness of great and lesser toe extension.^1^ The superior extensor retinaculum (SER) is a transverse band of tissue that houses extensor hallucis longus, extensor digitorum longus, and peroneus tertius tendons as well as the DPN and anterior tibial vessels.^2^ Pathophysiology of the condition involves elevated pressures deep to the SER causing neurologic and muscular compromise.^3^

The condition was initially described by Mubarak.^1,3^ Although there are few cases of ERS in the literature, it is speculated to be a progressive condition akin to acute carpal tunnel syndrome. Timely release of the SER has been identified as leading to prompt symptomatic resolution and restoration of function.

The natural history of ERS poorly understood and the condition itself is likely often missed. It is speculated that, if unrecognised, SER could result in ischaemic contracture of EHL, DPN sensation loss and loss of function of extensor digitorum brevis and extensor hallucis brevis.^3^ The 2 previously published case series on ERS associate the condition predominately with tibial physeal injuries, high-energy pilon injuries, and bimalleolar ankle fractures with a Weber C pattern. However, ERS is not typically associated with low-energy stable ankle injuries.

## The Case

A 33-year-old woman sustained an ankle fracture after slipping on tiles. She was managed conservatively initially following orthopaedic consultation. Two days later, she re-presented describing severe pain within 24 hours of the injury, with subsequent loss of sensation in her foot and inability to move her toes or perform dorsiflexion of her ankle. She described no pain at the time of presentation, however, with her main concern being her insensate foot. She had no significant medical history and was a non-smoker.

**Figure 1.**
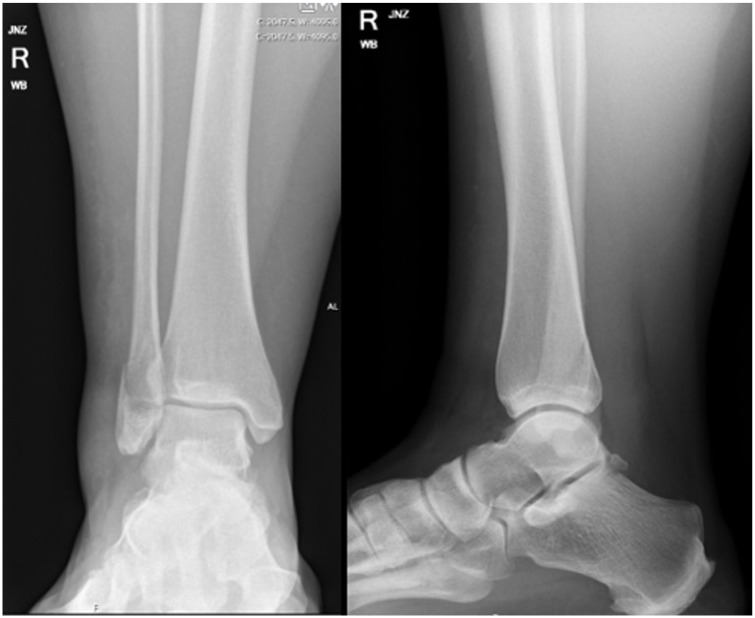
Anterior-posterior and lateral ankle weightbearing radiographs demonstrating a minimally displaced distal fibula fracture.

On examination, she had a large body habitus, marked midfoot and ankle swelling, absent superficial peroneal nerve (SPN) or deep peroneal nerve (DPN) sensation, and no active movement of hallux, lesser toes or ankle. She did not have pain on passive stretch of her toes, but remained tender over her fibula fracture. Plain radiographs showed a stable Weber B distal fibula fracture (Figure 1). Compartment pressures were not performed.

After no improvement in her clinical status with elevation and regular ice application overnight, a differential diagnosis of extensor retinaculum syndrome was considered and she underwent a release of her superior extensor retinaculum. A time of 40 hours had passed between onset of ERS symptoms and surgical release.

**Figure 2.**
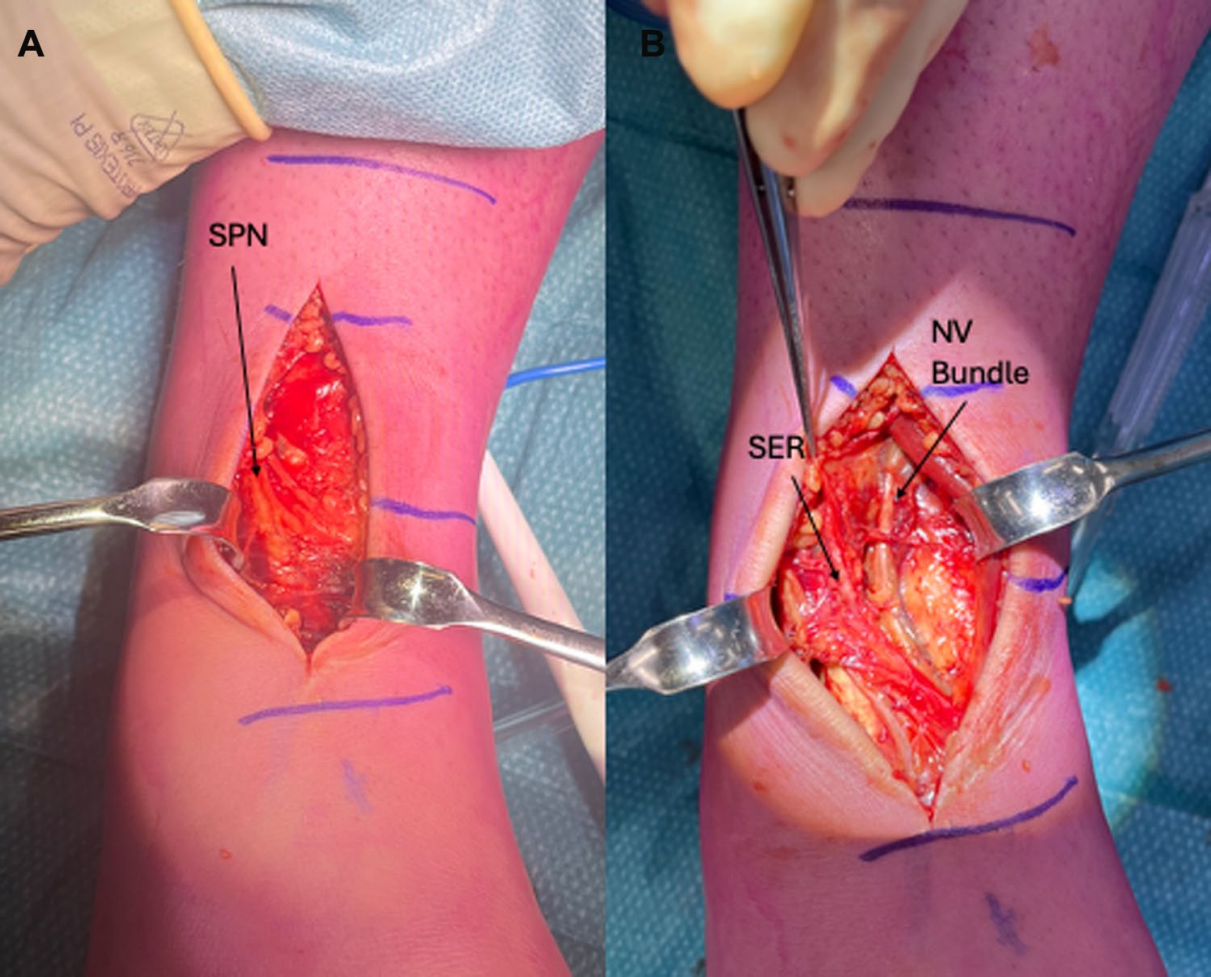
(A) Intra-operative photographs demonstrating the superficial peroneal nerve (SPN) with a haematoma surrounding it as it forms its terminal branches. (B) Release of the superior extensor retinaculum (SER), exposing the neurovascular bundle.

An anterior approach to the ankle was utilised. Intra-operative findings revealed a haematoma surrounding her superficial peroneal nerve (Figure 2), which was removed. Her SER was released with no obvious structural damage to her neurovascular bundle.

The patient had immediate post-operative return of SPN and DPN sensation, although motor function of her toes or ankle remained absent. A radiograph and a magnetic resonance image of her knee excluded a compressive injury or proximal fibula fracture. She was managed with an ankle-foot orthosis and monitored. Eighteen days post-operatively, nerve conduction studies were performed. Deep peroneal nerve motor action potential response was recorded using submaximal stimulation because of the patient’s pain, but nonetheless, was present. All other nerve conduction studies were within normal limits. At 8 weeks post-operatively, she had complete return of SPN and DPN sensation as well as motor function of her hallux, lesser toes, and ankle.

## Discussion

Our patient presented following a low-energy trauma with rapid onset of severe ankle pain over the following 24 hours and subsequently DPN sensory disturbance and loss of motor function of the greater and lesser toes. These symptoms were consistent with ERS; nevertheless, the additional SPN sensory disturbance and their inability to flex their toes could not initially be explained. The patient was free of pain at the time of her presentation and so it is speculated the window for eliciting pain on passive stretch of her EHL was missed, raising concern for a “missed ERS.” Although ERS did not account for the SPN symptoms initially, intraoperative findings of a haematoma compressing the SPN provided a plausible explanation.

The low-energy mechanism and stable fracture pattern made ERS an unlikely diagnosis as it has typically been associated with pediatric transitional fractures or high-energy foot and ankle injuries. Mubarak^1^ presented 6 paediatric patients with tibial physeal injuries who clinically presented with extensor retinaculum syndrome, intramuscular pressure >40 mm Hg deep to the superior extensor retinaculum, and had prompt relief of symptoms after release of the SER, with 2 patients experiencing residual DPN numbness. Despite this patient’s delayed presentation, they had complete return of DPN sensation after release of the SER.

Carlson et al^3^ provided the largest contemporary retrospective case series, spanning 18 years, which discussed 7 cases of adult ERS that occurred in high-energy mechanisms or post-operative setting after ankle fixation. All patients had both tibia and fibula fractures, and mechanism of injuries included motor vehicle accidents, roller derby, and ice skating. In contrast, our case involved a minimally displaced Weber B fibula fracture following a fall from standing height. Carlson’s group theorized that the natural history of ERS may involve ischaemic changes to EHL and long-term hypoaesthesia to the first web space. A cadaveric study has suggested the EHL is susceptible to compression and ischaemia in the setting of elevated pressure below SER, leading to weakness of toe extension.^4^ Despite their delayed presentation, our patient had restoration of DPN sensation immediately post-operatively and EHL function at 8-week clinical review.

## Conclusion

To our knowledge, this is the first reported case of an ERS in the setting of a stable Weber B fibula fracture. Surgical release of the superior extensor retinaculum and removal of the haematoma provided prompt relief of the patient’s symptoms and allowing for a good functional outcome. This case highlights that surgeons should consider ERS even in low-energy ankle trauma, and it is possible that this is a frequently missed diagnosis. Prompt identification and surgical intervention is required to optimise patient outcomes.

## Supplemental Material

sj-pdf-1-fao-10.1177_24730114251387694 – Supplemental material for Peroneal Nerve Palsy After a Minimally Displaced Weber B Fibula Fracture: A Rare Case of Extensor Retinaculum Syndrome Supplemental material, sj-pdf-1-fao-10.1177_24730114251387694 for Peroneal Nerve Palsy After a Minimally Displaced Weber B Fibula Fracture: A Rare Case of Extensor Retinaculum Syndrome by William Collins, Nikolas Drobetz and Cyrus Mehta in Foot & Ankle Orthopaedics
